# Histone Methylation and Chromatin Remodeling in Non-Small Cell Lung Cancer: Mechanisms of Oncogenesis and Emerging Therapeutic Strategies

**DOI:** 10.3390/biomedicines14071529

**Published:** 2026-07-08

**Authors:** A. Josephine Thrasher, Omar Bushara, Amy Gladstein, Katherine R. Doerig, Keren Adler, John Nathaniel Diehl, Sunil Singhal

**Affiliations:** 1 Department of Surgery, Hospital of the University of Pennsylvania, Philadelphia, PA 19104, USA; josephine.thrasher@pennmedicine.upenn.edu (A.J.T.);; 2 Department of Cancer Biology, Perelman School of Medicine, University of Pennsylvania, Philadelphia, PA 19104, USA

**Keywords:** NSCLC, methylation, epigenetic modifiers

## Abstract

Lung cancer remains the leading cause of cancer-related death, and, despite significant advancements in targeted therapy and immunotherapy, survival for patients with advanced non-small cell lung cancer (NSCLC) remains poor. An emerging area of interest is the role of epigenetic modifiers in both the pathogenesis and treatment of NSCLC. Herein, we review a selected group of chromatin-modifying genes implicated in NSCLC, organized by their function as writers (*KMT2A*, *SETD2*, and *EZH2*), erasers (the *KDM2*, *KDM5*, and *KDM6* demethylase families), and readers (the SWI/SNF subunits *SMARCA4* and *ARID1A*). Writers deposit activating or repressive marks on histones to regulate gene transcription, erasers remove these marks, and readers reposition nucleosomes and control DNA accessibility. Dysregulation of these genes has been associated with tumor proliferation, metastasis, treatment resistance, and altered response to immune checkpoint blockade in NSCLC. Research within this topic is emerging, and these genes represent promising potential therapeutic avenues as well as potential biomarkers. Finally, we review the clinical trials involving targeting these genes available in the current literature. The number of NSCLC-specific trials remains limited, with the most active development in *SMARCA2* inhibitors for *SMARCA4*-mutated tumors and *EZH2* inhibitors given in tandem with PD-1 blockade. We hope this review is hypothesis-generating for ongoing investigation into the role of epigenetic modifiers in NSCLC and their potential to expand the therapeutic armamentarium available for this disease.

## 1. Introduction

Non-small-cell lung cancer (NSCLC) is one of the most frequently diagnosed cancers and the leading cause of cancer deaths worldwide [1,2]. NSCLC is responsible for ~85% of lung cancers and includes common subtypes like adenocarcinoma (LUAD) and squamous cell carcinoma (SCC). Tobacco smoking is the biggest risk factor for developing lung cancer, but the incidence of lung cancer among non-smokers is rising globally [3].

NSCLC diagnosis is associated with high rates of morbidity and mortality, but current treatments still have low 5-year survival rates. Surgical resection is the foundation of treatment, with the addition of chemotherapy, radiotherapy, and targeted therapies depending on tumor size and stage [4,5]. Recent advancements in lung cancer treatment like nanodrug delivery, molecular targeted therapy, and immunotherapy have shown promise for improving survival rates in the future, but there is still much to be discovered about NSCLC, especially in light of treatment resistance [4,5,6]. Next-generation sequencing has accelerated the capabilities of identifying targets for new therapies, especially rarer genes or genes canonically associated with pathways unrelated to oncogenesis [7].

More recently, epigenetic modifiers have become a focus of promising research, both as biomarkers and possible drug targets [8]. Epigenetic modifiers, such as DNA methyltransferases, histone-modifying enzymes, and members of chromatin remodeling complexes, are often dysregulated in cancer, thereby offering an opportunity for identifying biomarkers, direct therapeutic targets, and possibilities for combination therapy [9] (Table 1).

In this review, we will discuss a selected group of chromatin remodeling genes that have been implicated in oncogenesis and/or tumor suppression, especially NSCLC. These genes belong to families of “writers” (enzymes that deposit epigenetic modifications), “readers” (proteins that recognize these modifications), and “erasers” (enzymes that remove epigenetic marks) [10]. The interactions between these three classes of epigenetic modifiers dictate a remarkable amount of dynamic chromatin remodeling, and there is a growing appreciation for their role in cancer development.

## 2. Writers

Writers are responsible for depositing methylation marks onto histones, which in turn modifies chromatin organization and thus gene expression [10,11]. In terms of gene expression, these methyl marks may be activating or repressive depending on the specific methyltransferase involved and the specific histone site being methylated. These methyltransferases have been characterized in the context of liquid tumors; however, the importance of mutations in genes encoding for these methyltransferases is being increasingly recognized in solid tumors, and specifically NSCLC. Below are summaries of the relevant literature in writers *KMT2A*, *SETD2*, and *EZH2*.

### 2.1. KMT2A

Lysine methyltransferase 2A, otherwise known as KMT2A or Mixed-lineage-leukemia 1 (MLL1), is part of the Trithorax group of epigenetic modifiers responsible for depositing activating trimethylation marks on histone 3 lysine 4 (H3K4me3) with their highly conserved SET domain (suppressor of variegation 3–9, enhancer of zeste and trithorax) [12,13]. This histone mark is notable for stabilizing transcription initiation and maintaining promoter accessibility and is implicated in many oncogenic processes [14]. *KMT2A* drives oncogenesis through a multifactorial process of fusion events that dysregulate transcription, enhancer architecture, and immune signaling. Though typically associated with hematologic malignancies due to the frequency of chromosomal translocations, *KMT2A* mutations have recently become more promising candidates for solid tumor research. Mutations in *KMT2A* in primary solid tumors can predict improved responses to immune checkpoint inhibitors, suggesting an immune regulatory role beyond oncogenic transcription [14]. In a pan-cancer analysis, *KMT2A* alteration was demonstrated to be associated with improved progression-free survival and overall response rate in patients receiving ICI, underscoring its potential utility as a biomarker [15]. However, this is a relatively rare mutation in solid tumors with a prevalence of around 5% [16]. Despite this, its association with improved response to ICI suggests this is a potentially fruitful area of future study [17].

Studies in the context of breast cancer have shown that *KMT2A* is involved in cell migration and metastasis in cancer, primarily through its control of actin filament assembly, which regulates protrusion and cell migration, as well as modulating cytokine signaling [18]. Current therapies targeting *KMT2A* dysregulation entail inhibition of cofactors like Menin and WDR5 [18]. Menin, unique to *KMT2A* and *KMT2B*, facilitates the trimethylation of H3K4 at bivalent promoters of developmental genes by providing a scaffold for KMT2A. Treating leukemias with Menin small-molecule inhibitors has been a promising avenue of treatment, which shows best efficacy when used in combination with other chemotherapy treatments [13,18]. WDR5, a cofactor recruited with *KMT2A* that promotes the assembly of the MLL/SET methyltransferase complex, is another potential therapeutic target for solid tumors like cholangiocarcinoma [14,18]. Outside of its interactions with *KMT2A*, WDR5 acts to recruit the oncoproteins N-MYC and c-MYC, amplifying the interest in developing WDR5 inhibitors for solid tumor treatment [19]. Given the existing foundation of using these cofactors as treatment targets for other cancers, these may serve as potential avenues in NSCLC.

Most solid tumor research has focused on the KMT3 family of methyltransferases, which are more associated with lung cancers and other solid tumors [11,20]. Though *KMT2A* is only altered in 5% of solid tumors, its role in locus-specific epigenetic activation in combination with recruitment of druggable cofactors like Menin and WDR5 makes *KMT2A* an interesting target for future solid tumor research [16].

### 2.2. SETD2

SETD2 is the sole histone 3 lysine 36 (H3K36) trimethyltransferase. H3K36me3 facilitates transcriptional activation, genome stability, and RNA splicing. Various loss-of-function mutations across the entire gene sequence of *SETD2* are found at a high frequency in different cancers [21]. Thus, this enzyme has been identified as a tumor suppressor. In solid tumors, it is more commonly found in renal cell carcinoma and gliomas, with mutations in up to 15–20% of tumors [22,23]. In lung adenocarcinoma (LUAD), *SETD2* is the most frequently mutated epigenetic modifier, with mutations in *SETD2* in 7–10% of cases [24,25,26,27]. Most cancer-causing mutations are found in the SET domain, which is the site of catalytic activity for its methyltransferase function. Loss of H3K36me3 due to *SETD2* inactivation showed an increase in proliferation, migration, invasion, and epithelial-to-mesenchymal transition (EMT) in LUAD, and both early- and late-stage tumors exhibited an accelerated progression of disease. Interestingly, in renal cell carcinoma, metastatic sites showed a higher rate of *SETD2* inactivation when compared with primary tumor sites [21]. This observation may support *SETD2* loss as a promoter of an aggressive disease phenotype.

In *KRAS*-driven LUAD, *SETD2* deficiency increases tumorigenesis, tumor burden, and lethality. Without H3K36me3 contributing to a closed chromatin state, loss of *SETD2* creates a more open epigenetic landscape, which leads to aberrant oncogene expression, thus accelerating tumor development [25,28]. However, *SETD2* inactivation in *KRAS*-driven LUAD also has unique treatment opportunities to pursue. Heightened mTORC1 signaling due to *SETD2* loss provides a targeted therapy option, and mouse models of LUAD with *SETD2* loss showed a reduction in tumor cell proliferation when mTORC1 signaling was blocked [24]. In another study, *SETD2*-deficient cells were found to have increased histone chaperone recruitment and were therefore more sensitive to inhibition of histone chaperones. Additionally, the upregulation of oncogenic transcription (required for tumor maintenance) due to *SETD2* deficiency makes cancer cells particularly sensitive to transcriptional inhibitors like dinaciclib, a CDK9 inhibitor [25].

In LUAD not driven by *KRAS*, *SETD2* deficiency still shows many opportunities for targeted therapies. *SETD2* appears to play an important role in DNA repair, DNA replication fork stability, and maintaining chromatin integrity. Therefore, compromising the function of *SETD2* in cancer can render the cancer cells more sensitive to radiation therapy [29,30]. Due to its role in repairing DNA, it has been suggested that PARP (Poly ADP-ribose polymerase) inhibitors could be a promising therapy for *SETD2*-deficient tumors [29].

### 2.3. EZH2

One of the most well-known groups of repressive chromatin modifying complexes is the Polycomb group, the key catalytic enzyme of which is EZH2 (enhancer of zeste homologue 2) [31]. EZH2 is the catalytic subunit of PRC2 (polycomb repressive complex 2), which trimethylates histone 3, lysine 27 (H3K27me3) to repress gene transcription. It functions as a master regulator of cell cycle progression, and dysregulation can accelerate cell proliferation and inhibit apoptosis [32]. *EZH2* is responsible for maintaining the epithelial state of cancer cells by repressing mesenchymal genes [33,34]. Direct and indirect inhibitors of *EZH2* have been trialed in different cell lines, with many showing decreased migration and invasion [32]. However, the efficacy of *EZH2* inhibition in attenuating cancer progression seems to depend on downstream compensatory mechanisms, as well as the specific driver mutation of the cancer type. *EZH2* can act to repress tumor suppressor genes, and while inhibition can therefore induce tumor suppression, it can also trigger compensatory epigenetic mechanisms to enhance tumor progression; indeed, in some studies *EZH2* inhibition can cause pro-tumor effects, or even increased micrometastasis [33,35].

Despite the paradoxical consequences of intervening with *EZH2* activity, there does seem to be utility in studying inhibition of *EZH2* as a therapeutic avenue for cancer. In LUAD, high expression of both *CBX2* (chromobox protein 2) and *EZH2* is positively correlated and also indicative of poor prognosis, but *CBX2* knockdown improved *EZH2* inhibitor therapy in a LUAD cell line [36]. Additionally, exploration of non-canonical functions of *EZH2* has yielded new opportunities for targeted therapies, as cancers like lung cancer depend on the non-catalytic functions of *EZH2* as well as its methyltransferase activity. *EZH2* interacts directly with the DNA methyltransferase family (DNMTs), and dual inhibition of DNMTs and *EZH2* was effective in treating both solid tumor and leukemia cells [37].

Despite its canonical function, *EZH2* levels do not directly correlate with H3K27me3 levels. It has been theorized that high EZH2 levels could actually signal a low PRC2 function, which can lead to a de-repression of the transcription factor forkhead box protein P2 (*FOXP2*)—a key promoter of migration and stemness [38]. This PRC2-deficient state can sensitize tumor cells to inhibitors of the bromodomain and extra-terminal domain (BET) family of proteins, which are epigenetic readers of acetylation marks to promote gene transcription. JQ1 is a small molecule inhibitor of bromodomain-containing protein 4 (BRD4), a member of the BET family, which is being investigated as part of a dual therapy with *EZH2* inhibitors to improve outcomes for tumors that would otherwise be resistant to *EZH2* inhibitors alone.

## 3. Erasers

Erasers are the counterparts of writers in that they remove methyl marks on histones. Again, depending on the context and specific methylation pattern, this can lead to either increased or decreased expression of associated genes [39,40,41]. The KDM family is a group of well-characterized erasers described below.

### 3.1. KDM Histone Demethylase Family

The KDM family of proteins are responsible for demethylating histones to regulate gene expression [39]. These epigenetic modifiers have been identified as potential therapeutic targets, as many KDMs are implicated in human disease [40]. The KDMs that contain a Jmj-C domain are of particular interest, as they are implicated in the pathologies of many diseases, including cancer [41]. While KDM5 and KDM6 are more established demethylases associated with cancer, KDM2 has recently become a viable candidate target for therapeutic research [41,42,43].

### 3.2. KDM2

KDM2A and KDM2B are Jmj-C-domain-containing histone demethylases responsible for demethylating lysine 36 on histone 3 (H3K36). They have critical roles in development, cell lineage commitment, and oncogenesis. Both polycomb group proteins and KDM2 demethylases, especially *KDM2B*, are commonly found to have mutations in human cancers as both tumor promoters and suppressors. *KDM2B* is strongly upregulated in different hematologic malignancies as well as pancreatic cancer, and a subset of NSCLC patients showed overexpression of KDM2A, representing poor prognosis [42]. *KDM2A* has been identified as a druggable target for lung cancer, as it is involved in alternative lengthening of telomeres and can have altered expression in lung cancer [43,44]. The demethylase activity of KDM2A was required for proliferation and invasion of *KDM2A*-overexpressing NSCLC cells in vitro, and subsequent knockdown decreased tumor invasion in mouse xenograft models [42]. In LUAD cell lines, knocking out *KDM2A* showed decreased tumor proliferation. Similarly, using the KDM2A inhibitor daminozide in two different LUAD cell lines showed an inhibitory effect on tumor proliferation [43].

Due to the identical nature of the demethylase domains in *KDM2A* and *KDM2B*, it is likely that inhibitors to one will inhibit the other, which can complicate the utility of small molecule inhibitors. Additionally, since the KDM2 family can act as both oncogenes and tumor suppressors in a highly context-dependent manner, inhibition can prove to be difficult. However, it appears that solid tumors would benefit the most from KDM2 inhibition, as hematologic malignancies are the main type of cancer that do not respond well to KDM2-targeted therapy [42]. One study identifies *KDM2A* as a therapeutic target not because of its oncogenic or tumor suppressor qualities, but because many cancer cells rely more heavily on genes that are not considered classical oncogenic drivers. This “non-oncogene addiction” can render cancer cells more sensitive to specific inhibition of non-oncogenes, like in the case of KDM2A [43]. These studies point towards canonical and non-canonical activities of the KDM2 family as relevant avenues for further research, particularly in NSCLC.

### 3.3. KDM5

KDM5 is a Jmj-C-domain containing demethylase that removes di- and tri-methyl groups from lysine 4 on histone 3 (H3K4me2, H3K4me3). There are four subfamily members: KDM5A, KDM5B, KDM5C, and KDM5D. *KDM5A*/*B* are found on autosomal chromosomes, whereas *KDM5C*/*D* are on the X and Y chromosomes, respectively. KDM5A and KDM5B are of particular interest as epigenetic regulators of cancer, since they are transcriptional regulators of differentiation and are found to be overexpressed in some cancers [45].

*KDM5A* works with critical tumor suppressor pRb to reinforce the silencing of E2F target genes via H3K4me3 demethylation, suggesting a tumor suppressive role. However, *KDM5A* knockdown and overexpression experiments have yielded mixed results, with some supporting the tumor suppressor role and others suggesting a counterintuitive oncogenic role that supports tumor development [45,46,47]. KDM5A has been shown to enhance immune response in cancers like NSCLC by modulating macrophage phenotype, but it is also implicated in chemoresistance and targeted therapy resistance by inhibiting other tumor suppressors [46].

*KDM5A* has significant sequence homology and functional similarities to KDM5B, but they have different target genes, which suggests context-dependent and sometimes paradoxical roles for cell cycle regulation and development [48]. *KDM5B* was first reported as an oncogene in breast cancer, but has since been identified in many other tumor types [46]. Overexpression of KDM5B has been associated with upregulation of the E2F/Rb pathway in lung cancer, and *KDM5B* has a relatively high number of mutations found in LUAD and SCC sequencing databases [47]. It has a negative association with overall survival, and potential mechanisms are related to its promotion of EMT and induction of stem-cell-like phenotype by the Zeb1/2 and c-MET pathways, respectively. *KDM5B* may also confer radioresistance in NSCLC cells by removing H3K4me3 that interferes with DNA repair factor recruitment, and it also confers chemoresistance to treatments like gefitinib in NSCLC [45].

The *KDM5C* and *KDM5D* genes are very similar, but *KDM5D* is located on the Y chromosome and has been described as a tumor suppressor, with *KDM5D* loss shown as a negative prognostic indicator in lung, gastric, and prostate cancer [46]. *KDM5C*, like *KDM5A*/*B*, has shown both tumor suppressor and oncogenic roles in a context-dependent fashion, but it is suggested to enhance immunotherapy response in NSCLC [46].

Creating targeted therapies to KDM5 subfamily proteins has proven difficult due to the similarities between the different proteins, as well as delivery mechanisms for KDM5 inhibitors, but it remains a promising area of future research [49].

### 3.4. KDM6

KDM6 is also a Jmj-C-domain containing demethylase that removes di- and tri-methyl groups from lysine 27 on histone 3 (H3K27me2, H3K27me3). There are three subfamily members: KDM6A, KDM6B, and KDM6C, though KDM6C remains mostly understudied due to its lack of enzymatic activity and structural redundancy with KDM6A [50]. As with other histone demethylases discussed above, KDM6 has many paradoxical reports of engaging in tumor suppressor activity as well as oncogene activity, especially in NSCLC [51]. Additionally, as H3K27me3 is such an abundant mark, altering levels of KDM6 can lead to compensatory epigenetic changes that are unpredictable, making targeted therapeutics a challenging puzzle [50].

*KDM6A*, otherwise known as *UTX*, is present on the X chromosome and has been profiled in many studies concerning different types of cancer. In NSCLC, it interacts with *KRAS* and E-cadherin in a tumor suppressor function in LUAD cell lines; however, it can also recruit *EZH2* and activate the Wnt/β-catenin/c-MYC pathway to promote NSCLC tumor progression [52,53]. *KDM6B* promotes cancer cell growth via the MAPK pathway, but is also induced by activation of the RAS/RAF pathway, which activates tumor suppressor proteins [51].

A recent study also found that the tumor driver *EGFR* may upregulate *KDM6A* through the JAK/STAT3 pathway, promoting tumor proliferation and migration in NSCLC, but the functional mechanism remains unclear [54]. Since histone demethylases have many roles ranging from canonical enzyme activity to chromatin scaffolding, there is great difficulty with assigning labels to oncogene activity or tumor suppressor activity since their function may be context-dependent. This also contributes to difficulty with creating targeted inhibitors, as KDM6 inhibitors are not subfamily-specific and could also affect KDM5 and other Jmj-C-domain containing demethylases [50].

Since KDM6 inhibitors may currently have limited utility due to their off-target effects, using them in tandem with *EZH2* inhibitors or mTOR inhibitors may be beneficial, as tumors deficient in *KDM6A* have high mTOR signaling activity [52]. Another common mutation in NSCLC is in *SMARCA4*, a catalytic subunit of the SWI/SNF complex, which leads to aberrant accumulation of H3K27me3 and could be a potential target for KDM6 inhibition [55]. Many of the properties of KDM6 subfamily proteins have yet to be discovered, but they undoubtedly play important roles in NSCLC and other cancers.

## 4. Readers

Readers are the final group of epigenetic modifiers and are responsible for modifying chromatin accessibility, in turn modifying gene expression. Chief among them are *SMARCA4* and *ARID1A*, which are relatively well-characterized in solid tumors, with mutations in the former being associated with aggressive biology [56,57]. Below, we summarize readers and the available clinicopathologic data associated with their alteration in solid tumors.

### 4.1. SWI/SNF Complexes

SWI/SNF is a group of chromatin remodeling complexes that use ATP to modify chromatin structure. Its name comes from screens in yeast (SWItching and Sucrose Non-Fermentable), but it is also found in mammals, sometimes referred to as BRG1-associated factors or BAF. By consuming ATP, SWI/SNF complexes can move nucleosomes to modify chromatin compaction and therefore transcriptional activity [56]. Studies have shown that SWI/SNF complex genes are commonly mutated in a wide variety of cancer types and up to 10% of LUAD, suggesting a tumor suppressor role [56,57,58]. Furthermore, cancers with SWI/SNF mutations may also depend more heavily on canonical and non-canonical activity of PRC proteins like EZH2, further demonstrating the interdependencies of epigenetic modifiers in human disease [57].

### 4.2. SMARCA4

SMARCA4 is one of the catalytic ATPase subunits of SWI/SNF, which regulates gene expression by altering chromatin structure and nucleosome position, and is one of the most frequently inactivated subunits of SWI/SNF in NSCLC [59]. It contains a bromodomain that recognizes acetylated lysine residues on histone tails, further playing a role in regulating gene transcription. In human cancers, *SMARCA4* is commonly co-mutated with *TP53*, *STK11*, *KEAP1*, and *KRAS*. For patients with NSCLC, *SMARCA4* mutations are present in 7–11% of cases and are associated with poor outcomes [60].

There are two types of *SMARCA4* mutations linked to epigenetic dysregulation: truncating mutations/loss-of-function, and missense mutations (suggesting dominant-negative or gain-of-function effects). Both of these mutation classes co-occur with other tumor driver mutations, with truncating/loss-of-function mutations having the most negative prognosis; however, this class of mutations has a better response to immune checkpoint inhibitor therapy [61].

*SMARCA4*-deficient tumors can manifest in different ways in NSCLC, with *SMARCA4*-deficient undifferentiated tumor (“*SMARCA4*-UT”) and thoracic sarcoma being two main groups. The *SMARCA4*-UTs are much more rare, and are not responsive to inhibition of *SMARCA2* that creates a synthetic lethality effect in other *SMARCA4*-deficient tumors [62]. In these *SMARCA4*-deficient tumors, protein expression, rather than specific gene mutations, seems to be a better indicator of prognosis [63].

*SMARCA4* mutations typically arise in patients with a history of smoking, but case studies have also described tumors found in patients with no smoking history [64,65]. Chemoimmunotherapy seems to perform best for treatment of these tumors [63]. Despite high rates of PD-L1 negativity, PD-L1 blockade seems to be beneficial, as well as inhibition of *CDK4/6, AURKA, ATR*, and *EZH2* in preclinical models [61]. Due to the severity of these tumors, it is clear that *SMARCA4* presents a unique opportunity for further investigation of potential targeted therapies.

### 4.3. ARID1A

Along with SMARCA4, ARID1A is one of the most frequently inactivated SWI/SNF subunits in NSCLC [59]. ARID1A is responsible for recruiting SWI/SNF to its target sequences through interactions with DNA and protein, and acts as a tumor suppressor that is mutated in different cancers, with the dominant mutation types being truncating mutations or splice-site mutations [66,67,68]. Loss of ARID1A protein expression due to mutation can serve as an effective prognostic marker, especially for LUAD, as loss of *ARID1A* expression is associated with poor prognosis [68].

Loss of *ARID1A* expression correlates with lymph node infiltration, metastasis, and increased TNM stage [69]. It has been suggested that *ARID1A* loss not only activates the PI3K/Akt pathway, but also activates receptor tyrosine kinases like EGFR and ERBB3 to promote metastasis and tumor proliferation [70]. Cell cycle-related proteins like cyclins and CDKs are also upregulated in *ARID1A* loss, which leads to accelerated cancer cell division in *ARID1A* knockdown experiments. These qualities make *ARID1A* a desirable candidate for a biomarker that can predict responsiveness to tyrosine kinase inhibitors in NSCLC driven by activating *EGFR* mutations [70].

In NSCLC patients with *ARID1A* mutations, treatment with PD-1/PD-L1/CTLA-4 inhibitors showed better outcomes, and SWI/SNF mutations in general correlated with better efficacy of ICI therapy [71,72]. These outcomes are promising for future investigation of *ARID1A* and its utility for guiding treatment outcomes. The influence of *ARID1A* on tumor immune microenvironment is still central to many ongoing studies, but it seems that *ARID1A* mutation can beneficially alter the tumor microenvironment to allow for a higher abundance of CD8+ T cells, and autophagy inhibitors may be a useful supplement for ICI therapy [66,73,74]. Further studies are needed to validate these combination therapies for NSCLC with *ARID1A* mutations, but it is a promising biomarker for guiding future treatment.

## 5. Implications for Future Therapies

Epigenetic modifiers provide an excellent avenue for exploring new opportunities for NSCLC treatment and potential biomarkers. Many of the genes identified in this review participate in common pathways, and since epigenetic marks are highly dynamic, perturbation of one chromatin remodeler can often influence another [10]. The most immediate treatment implications for these targets will likely be to incorporate them as adjunctive treatments along with existing regimens, as they can serve as modulators of the immune system and the cell cycle [75,76]. Identifying mutations in these genes can allow for the addition of small molecule targeted inhibitors to enhance the function of conventional therapies and immune checkpoint inhibitors [77,78]. *SETD2* mutations appear to indicate vulnerability in the setting of treatment with PARP inhibitors [79], whereas *SMARCA4-* and *ARID1A*-mutated tumors seem to be particularly vulnerable to immune checkpoint inhibitors [66,71]. *KDM6* and *SMARCA4* mutations are also suggested to be responsive to EZH2 inhibitors [57,80], and small molecule inhibitor JQ1 is a potential treatment for *EZH2*-mutated tumors [81,82]. The interdependence of these different epigenetic modifiers may be viewed as a hurdle for limiting off-target effects, but also an opportunity to better understand the epigenetic ecosystem of tumor cells.

## 6. Clinical Trials

The clinical translation of epigenetic-targeted therapy in NSCLC remains in its early stages, and there has yet to be strong evidence supporting any of these mutations as a bona fide target. A summary of existing trials is found within Table 2, although the majority have limited data at this juncture. The results will be followed with great interest as potentially promising adjuncts to the current standard of care for NSCLC. The most active area of clinical development is in *SMARCA4*-mutant NSCLC, where two SMARCA2-selective agents are currently being studied, with potential efficacy based on the principle of synthetic lethality. PRT3789, an intravenous SMARCA2 degrader (NCT05639751) [83] and FHD-909, an oral SMARCA2-selective inhibitor (NCT06561685) [84], are both being evaluated in phase I trials for *SMARCA4*-mutant NSCLC patients, with early data demonstrating some clinical activity in heavily pretreated patients. EZH2 inhibition has been trialed in NSCLC in a phase Ib/II trial evaluating tulmimetostat in combination with pembrolizumab in patients who have progressed on prior therapy (NCT05467748) [85]. Patients with *ARID1A*-mutant NSCLC are eligible for an ongoing phase II trial of tazemetostat in *ARID1A*-mutant solid tumors (NCT05023655) [86]. Other targets discussed in this review have not been trialed in NSCLC, although early trials in other tumor types have been mixed. Overall, the available evidence is in its infancy and is limited, and additional trials are warranted to determine which of these targets will translate into durable clinical benefit for patients with NSCLC (Table 2).

## 7. Current Testing Paradigms and Future Directions

The current standard of care for molecular diagnosis in NSCLC is next generation sequencing (NGS) of the surgical specimen, which tests for hundreds of cancer-associated genes at once and detects both common drivers and rarer, understudied mutations [91,92,93]. Routine use of NGS has been especially valuable for the study of rare genes, including the epigenetic modifiers discussed in this review, and allows them to be linked to clinicopathologic presentation and outcomes [26,27]. A potential advancement to the current testing paradigm is performing NGS on biopsy specimens rather than the resection specimens, which would establish the molecular diagnosis preoperatively and allow potentially targeted neoadjuvant therapy, building on the survival benefit of neoadjuvant chemoimmunotherapy in resectable NSCLC [94]. A further advancement is NGS of circulating tumor DNA (ctDNA), which detects tumor-derived mutations from a peripheral blood draw rather than tissue [95]. Because it requires only blood, ctDNA could extend molecular diagnosis to patients whose tumors are difficult to biopsy and could be sampled serially to monitor residual disease and treatment response over time. The same minimally invasive approach would also facilitate research into genes such as the epigenetic modifiers reviewed here, allowing their mutations and dynamics to be studied across large patient cohorts without dependence on tissue specimens.

## 8. Conclusions

We hope that this review can be hypothesis-generating for future study on epigenetic modifiers and their role in lung cancer. Further research is required to fully elucidate the role of these chromatin regulators in oncogenesis, with significant potential for both basic and translational studies. Additionally, these genes can be leveraged for both the development of novel cancer treatments as well as biomarkers for treatment response. Both preclinical studies and clinical trials will be needed to fully define the benefit of personalizing cancer treatment approaches to incorporate these epigenetic alterations [96]. However, the current literature supports promise in the study of these epigenetic modifiers as actionable mutations that may one day inform treatments for patients with NSCLC.

## Figures and Tables

**Table 1:** Epigenetic Modifiers of NSCLC Oncogenesis.

Gene	Function	Role in NSCLC Oncogenesis
*KMT2A*	Writer; H3K4 methyltransferase	Drives transcription, cell migration, and metastasis via actin filament assembly and cytokine signaling
*SETD2*	Writer; H3K36 trimethyltransferase	Most frequently mutated epigenetic modifier in LUAD; loss accelerates tumorigenesis, particularly in KRAS-driven LUAD
*EZH2*	Writer; H3K27 trimethyltransferase	Frequently overexpressed in NSCLC; promotes proliferation and immune evasion; correlates with poor prognosis
*KDM2A/B*	Eraser; H3K36 demethylase	Overexpressed in some NSCLC subsets; promotes proliferation and invasion; associated with poor prognosis
*KDM5A/B*	Eraser; H3K4me2/3 demethylase	Context-dependent oncogenic and tumor suppressor roles; KDM5B promotes dedifferentiation and confers chemo/radioresistance
*KDM6A/B*	Eraser; H3K27me2/3 demethylase	Paradoxical oncogene/tumor suppressor activity; interacts with KRAS and Wnt/β-catenin pathways
*SMARCA4*	Reader; SWI/SNF catalytic subunit	Mutated in up to 10% of NSCLC; often co-mutated with TP53, STK11, KEAP1, KRAS; poor prognosis
*ARID1A*	Reader; SWI/SNF DNA-binding subunit	Frequently inactivated in NSCLC; activates PI3K/Akt and EGFR/ERBB signaling; correlated with advanced stage

**Table 2:** Active Clinical Trials Relating to Epigenetic Modifiers of Disease.

Gene	Intervention	Trial Phase	Indication	Trial Number
*EZH2*	Tulmimetostat + pembrolizumab	Ib/II	Advanced NSCLC	NCT05467748 [85]
*EZH2/ARID1A*	Tazemetostat	II	ARID1A-mutant solid tumors	NCT05023655 [86]
*EZH2/SMARCA4*	Tazemetostat + nivolumab + ipilimumab (TAZNI)	I/II	Pediatric SMARCA4-deficient tumors	NCT05407441 [87]
*SMARCA4*	PRT3789 ± docetaxel (SMARCA2 degrader)	I	SMARCA4-mutated solid tumors, NSCLC-enriched	NCT05639751 [83]
*SMARCA4*	FHD-909/LY4050784 (SMARCA2 inhibitor)	I	SMARCA4-mutant solid tumors, NSCLC primary	NCT06561685 [84]
*KMT2A*	Revumenib ± chemotherapy	I/II	Colorectal and other solid tumors	NCT05731947 [88]
*SETD2*	Adavosertib (WEE1 inhibitor)	II	SETD2-mutated solid tumors	NCT03284385 [89]
*KDM6A*	Pemetrexed	II	Metastatic urothelial/solid tumors with KDM6A or KMT2D mutation	NCT06630416 [90]

## Data Availability

No new data were created or analyzed in this study.
